# Correction to “Development of a Novel Functional Yogurt Fortified With *Ziziphus lotus* Fruit: Enhancement of Antioxidant Capacity, Physicochemical Properties, and Sensory Quality”

**DOI:** 10.1155/ijfo/9784834

**Published:** 2026-06-27

C. Hamdi, O. Amri, H. Elmourabit, et al., “Development of a Novel Functional Yogurt Fortified With *Ziziphus lotus* Fruit: Enhancement of Antioxidant Capacity, Physicochemical Properties, and Sensory Quality,” *International Journal of Food Science*, 2026, 2895245, https://doi.org/10.1155/ijfo/2895245

In the article, Khadija Elmehrach should have been listed as a co‐corresponding author. This has been corrected in the article.

We apologize for this error.

